# Herbal preparation (HemoHIM) enhanced functional maturation of bone marrow-derived dendritic cells mediated toll-like receptor 4

**DOI:** 10.1186/s12906-016-1045-9

**Published:** 2016-02-19

**Authors:** Sung-Ju Lee, Jong-Jin Kim, Kyung-Yun Kang, Yun-Ho Hwang, Gil-Yeon Jeong, Sung-kee Jo, Uhee Jung, Hae-Ran Park, Sung-Tae Yee

**Affiliations:** Department of Pharmacy, Sunchon National University, 255 Joongang-Ro, Seokhyeon-Dong, Suncheon, 549-742 Republic of Korea; Department of Chemistry, National University of Singapore, Singapore, 117543 Singapore; Radiation Research Division for Bio-Technology, Advanced Radiation Technology Institute, Jeongeup Campus of Korea Atomic Energy Research Institute (KAERI), Jeongeup, Republic of Korea

**Keywords:** Herbal Composition (HemoHIM), Bone Marrow-Derived Dendritic Cells, Toll-Like Receptor 4 (TLR4), CD4^+^ T cells, CD8^+^ T cells

## Abstract

**Background:**

HemoHIM, which is an herbal preparation of three edible herbs (*Angelicam gigas* Nakai, *Cnidium offinale* Makino, and *Peaonia japonica* Miyabe), is known to have various biological and immunological activities, but the modulatory effects of this preparation on dendritic cells (DCs)-mediated immune responses have not been examined previously. DCs are a unique group of white blood cells that initiate primary immune responses by capturing, processing, and presenting antigens to T cells.

**Results:**

In the present study, we investigated the effect of HemoHIM on the functional and phenotypic maturation of murine bone marrow-derived dendritic cells (BMDCs) both in vitro and in vivo. The expression of co-stimulatory molecules (CD40, CD80, CD86, MHC I, and MHC II) and the production of cytokines (IL-1β, IL-6, IL-12p70, and TNF-α) were increased by HemoHIM in BMDCs. Furthermore, the antigen-uptake ability of BMDCs was decreased by HemoHIM, and the antigen-presenting ability of HemoHIM-treated mature BMDCs increased TLR4-dependent CD4^+^ and CD8^+^ T cell responses.

**Conclusions:**

Our findings demonstrated that HemoHIM induces TLR4-mediated BMDCs functional and phenotypic maturation through in vivo and in vitro. And our study showed the antigen-presenting ability that HemoHIM-treated mature BMDCs increase CD4^+^ and CD8^+^ T cell responses by in vitro. These results suggest that HemoHIM has the potential to mediate DC immune responses.

## Background

Dendritic cells (DCs) are the immune cells that are responsible for the presentation of antigens to T cells. The main functions of DCs are to capture and present antigens on their surfaces and thus activate other immune cells. DCs are the most potent antigen presenting cells (APCs) [1], originate from the bone marrow, and play a pivotal role in the induction of adaptive immunity as initiators of T cell responses against pathogens and tumors [2–5]. DCs are found in the peripheral blood of tissues as immature DCs and are classified as immature or mature DCs. Immature DCs activate T cells weakly but efficiently capture antigens associated with pathogens, bacteria, tumors, and inflammatory cytokines and then begin to mature and migrate to lymph nodes [3, 5–7]. Mature DCs have lower antigen uptake abilities than immature DCs but express higher levels of co-stimulatory molecules and major histocompatibility complex class (MHC) I and II on their surfaces [1, 8]. These cells play key roles in the antigen-specific T cell responses that are required to initiate adaptive immune responses [2, 3, 9]. In particular, mature DCs induce the activation of helper-T cells, cytotoxic-T cells and cell-mediated immune responses and enhance the anti-tumor effects of cytotoxic-T cells [10]. Recent research reveals the development of DC-based anti-tumor immunotherapy, which is driven by the strong interaction between DCs and T cells, whereby DCs present tumor antigens via MHC I and MHC II and thus activate tumor-specific- CD8^+^ and CD4^+^ T cells [10–12]. Like APCs and other immune cells, DCs express specific repertoires of Toll-like receptors (TLRs), which are capable of recognizing microbial components [7, 10, 13], binding to corresponding ligands, and triggering signaling pathways that induce DC activation [7, 10, 13]. TLRs have been reported to be the key receptors responsible for recognizing specific components of antigens [14]. Of the various TLRs, TLR-2 and TLR-4 are particularly important markers of DC activation [15–17], and participate in innate defense against bacterial infections [15, 18–20]. Furthermore, these receptors have been implicated in the activation of DCs by exogenous and endogenous adjuvants [12], and TLR-4 usually induces Th1 activation. [10].

HemoHIM is a well-known herbal mixture that consists of consisting of Angelica Radix, Cnidii Rhizoma, and Paeonia Radix [21–31] and has been reported to inhibit various activities of human mast cells [23, 24], to increase the secretion of IFN-γ and IL-2, to decrease the secretion of IL-4 by the spleen and lymphocytes [24, 25], to improve immune function [21, 24], to exert anti-inflammatory effects on carrageenan-induced edema [21], to ameliorate oxidative stress, such as stress induced by irradiation [26], and to affect the activation of immune cells [27]. In addition, HemoHIM has been reported to act as an immune-modulatory agent [28–30], to have anti-tumor effects [31], and to rescue white blood cells and lymphocytes exposed to ionizing radiation (IR) [21].

In this study, we investigated whether HemoHIM enhances the functions of DCs for potential applications in DC-based anti-tumor therapy. In particular, we investigated the HemoHIM-induced TLR4-mediated functional and phenotypic maturation of bone marrow-derived dendritic cells (BMDCs) and the efficiency of antigen-presentation by these cells to CD4^+^ T cells and CD8^+^ T cells.

## Methods

### Animals and experimental treatments in vivo

Female 8- to 12-week-old C57BL/6 mice, weighing 20-22 g, were purchased from Orientbio (Orientbio Inc., Iksan, Korea). Female 8- to 12-week-old BALB/c mice, weighing 20-22 g, were purchased from DAE-HAN Biolink (Eumseong, Korea). Male 8- to 12-week-old C57BL/6 wild-type, TLR2-deficient, and TLR4-deficient mice were obtained from Dr. Park (College of Medicine, Konyang University, Daejeon, Korea). The animals were housed in a controlled environment [22 ± 2 °C and 50 ± 5 % (relative humidity)] in polycarbonate cages, and fed a standard animal diet with water. For in vivo experiments, mice were randomly divided in to 2 groups of 6 animals. Control group was orally administered with sterilized distilled water (D.W) alone (200 μl/mice) once a day for 4 weeks. And Treatment group was orally administered with HemoHIM (100 mg/kg) in D.W (200 μl/mice) once a day for 4 weeks [21, 32]. All mice were treated in strict accordance with the guidelines issued for the care and use of laboratory animals by the Sunchon National University Institutional Animal Care and Use Committee (SCNU IACUC). All procedures were approved by the SCNU IACUC (Permit Number: 2013-1).

### Reagents and antibodies

Recombinant mouse granulocyte-macrophage colony-stimulating factor (GM-CSF) and interleukin (rmIL)-4 were purchased from R&D Systems (Minneapolis, MN, USA), Dextran-FITC (40,000 Da), propidium iodide (PI), ovalbumin (OVA), and mitomycin C (MMC) were purchased from Sigma-Aldrich (Steinheim, Germany), and carboxyfluorescein succinimidyl ester (CFSE), lipopolysaccharide (LPS), and Pam3CSK4 (Pam3) were purchased from Invitrogen (Carlsbad, CA, USA). The following FITC- or PE- conjugated monoclonal antibodies (Abs) and non-labeled Abs were purchased from BD Biosciences (San Jose, CA, USA) : FITC-annexin V, CD16/32 (2.4G2), CD11c (HL3), CD40 (HM40–3), CD80 (16–10A1), CD86 (GL1), H-2Kb (AF6-88.5), I-A[b] (AF6–120.1), and CCR7 (4B12). Cytokine ELISA primary and secondary -antibodies specific for murine IL-1β, IL-6, IL-12p70, IFN-γ, IL-4 and TNF-α were purchased from BD Biosciences (San Jose, CA, USA), and the OT-1 peptide (OVA_257-264_) was purchased from Invivogen (San Diego, CA, USA). CD4^+^ T cell isolation kit II, CD11c Isolation Kit II and Separation Columns were purchased from MACS Miltenyi Biotec (Auburn, USA). 5-Bromo-2′-Deoxy-Uridine Labeling and Detection Kit III were purchased from Roche (Salt Lake City, UT, USA).

### Preparation of HemoHIM

Equal amounts of the three edible medical herbs [i.e., Angelica Radix (roots of *Angelica gigas Nakai*), Cnidii Rhizoma (roots of *Cnidium offinale Makino*), and Paeonia Radix (roots of *Paeonia japonica Miyabe*)] were mixed at 1:1:1 ratio. To obtain the total extract (HIM-I), this mixture was decocted for 4 h in boiling water. And then, the total extract (HIM-I) was divided into two parts. The ethanol-insoluble polysaccharide fraction was obtained by from one part of total extract (HIM-I) by precipitation in 80 % (vol/vol) ethanol and then added to the other part of total extract (HIM-I), freeze-dried, and stored at −20 °C (HemoHIM). HemoHIM was composed of carbohydrates (60.4 %), protein (6.0 %), and other components (33.6 %) and other including polyphenols (33.6 %), (data not shown). The immune modulating components in HemoHIM were the ethanol-insoluble fraction, the polysaccharide content was 40.9 % ±3.8 (data not shown) and other carbohydrate components in HemoHIM were acidic-polysaccharide ethanol soluble fraction, the polysaccharide content of which was 19.5 % (data not shown). In addition, the functional component analysis in the ethanol-soluble fraction of HemoHIM was performed via high-performance liquid chromatography (HPLC) using phytochemicals, as follows: gallic acid [0.2 % (±0.06)], chlorogenic acid [0.33 % (±0.05)], paeoniflorin [1.32 % (±0.15)], nodakenin [0.58 % (±0.04)], and benzoic acid [0.17 % (±0.05)]. In particular, these herbs are suggested as raw materials in the Korean Food Code. Finally, HemoHIM has been proven to be safe for long-term administration [21, 24–26, 29–34].

### Generation and activation of bone marrow-derived DCs

BMDCs were obtained from the femurs and tibias of C57BL/6 mice, and red cells were treated with lysis buffer solution (4.15 g of ammonium chloride per 500 mL of 0.01 M Tris–HCl buffer). The cells were washed and cultured in six-well tissue culture plates at 1 × 10^6^ cells/mL in complete RPMI culture medium supplemented with 10 % fetal bovine serum (FBS), 2-mecaptoethanol (50 μM/mL), IL-4 (1,000 U/mL), and GM-CSF (1,000 U/mL). The culture medium was changed on culture days 2 and 4. New medium and cytokines (i.e., GM-CSF and IL-4) were added after flushing out non-adherent cells. On day 6, loosely adherent clustered cells were defined as immature BMDCs, treated with LPS and HemoHIM for 24 h and then harvested.

### Isolation and activation of spleen-DCs

Mice were orally administered daily for 4 weeks. After 4 weeks, spleen-DCs (s-DCs) from splenocytes suspensions of C57BL/6 mice were isolated using the CD11/c^+^ isolation kit II and Separation Columns according to the manufacturer’s instruction. Purity was greater than 94 %. S-DCs and treated with lipopolysaccharide (LPS, 100 ng/ml). After 24 h incubation, we analyzed co-stimulatory of s-DCs using the flow cytometory, and measured cytokine production with ELISA assay [35–43].

### Cytotoxicity assay

Apoptosis induction by HemoHIM was quantified via flow cytometry using Annexin V-FITC and a propidium iodide (PI) solution, according to the manufacturer’s instructions. Briefly, BMDCs (1 × 10^6^/well) were seeded onto 24-well plates and exposed to HemoHIM or LPS for 24 h. Apoptosis was quantified by staining with Annexin V-FITC/PI. Finally, the samples were analyzed by flow cytometry, and apoptosis percentages were calculated by counting Annexin V-positive cells.

### Flow cytometry

After incubation for 24 h, DCs were harvested, washed with fluorescence-activated cell sorting (FACS) solution buffer, and blocked with anti-mouse CD16/32, which blocks FcγII and FcγIII receptors on DCs, for 30 min on ice. After blocking, the cells were stained with fluorescence-labeled antibodies (i.e., anti-mouse CD11c-FITC, CD40-PE, CD80-PE, CD86-PE, MHC I-PE, or MHC II-PE) for 30 min on ice, washed with FACS solution buffer, and analyzed via flow cytometry. Data analysis was performed using the BD FACS Diva software.

### Antigen uptake assay

To assess the antigen uptake activity of BMDCs, on culture day 6, the BMDCs were treated with HemoHIM (100 μg/ml) and LPS (1 μg/ml) or left untreated for 24 h. Then, 2 × 10^5^ of the obtained mature-BMDCs were incubated at 37 °C for 2 h with 1 mg/mL FITC-dextran, washed with FACS solution buffer, and analyzed via flow cytometry.

### OVA-specific Th1 cell preparation

The mice were immunized intraperitoneal injection (i.p.) with incomplete Freund’s adjuvant (IFA) or complete Freund’s adjuvant (CFA) (Pierce) and immunized with OVA on day 0 (OVA-IFA) and on day 7 (OVA-CFA). Spleens were harvested from OVA-immunized mice on day 14. CD4^+^ T cells were isolated from the spleens using a CD4^+^ T cell Isolation Kit II and then activated with OVA (100 μg/ml) and syngeneic APCs. OVA-specific CD4^+^ T cells were selected by limiting dilution, and OVA-specific T (CD4^+^, IFN-γ^+^) cells were counted using a FACScanto II [10, 44, 45].

### Proliferation assay of OVA-specific Th1 cells

To investigate the antigen-presenting ability of OVA or OVA-pulsed HemoHIM BMDCs, 3 × 10^4^ cells were incubated with 5 × 10^4^ OVA-specific Th1 cells for 24 h or 48 h in 200 μl of culture medium in 96-well cell culture plates. Also, to investigate the antigen-presenting ability of OVA-pulsed s-DCs of group with oral administered HemoHIM, 3 × 10^4^ s-DCs of orally administered group (D.W or HemoHIM) were incubated with 5 × 10^4^ OVA-specific Th1 cells for 24 h or 48 h in 200 μl of culture medium in 96-well cell culture plates. Proliferation was evaluated using a 5-bromo-2′-deoxy-uridine Labeling and Detection Kit III. Cytokine levels were determined via ELISA. The results are expressed as the mean of experiments performed in triplicate.

### Mixed-lymphocyte reaction (MLR) assay

CD4^+^ T cells from splenocyte suspensions from BALB/c mice were isolated using a CD4^+^ T cell isolation kit II, and the purity was greater than 95 %. On culture day 6, BMDCs were incubated with or without HemoHIM and LPS for 24 h. Mature BMDCs were harvested, treated with 50 μg/ml of MMC for 25 min, and then washed. OVA_257-264_-specific CD8^+^ T cells were obtained from splenocyte suspensions from C57BL/6 OT-1 T-cell receptor (TCR) transgenic mice. CD4^+^ T cells were co-cultured with BMDCs, and CD4^+^ T cell proliferation was determined using a 5-bromo-2′-deoxy-uridine Labeling and Detection Kit III. In addition, cytokine secretion by CD4^+^ T cells was determined using an ELISA. The results are expressed as the mean of triplicate experiments. OVA_257-264_-specific CD8^+^ T cells were washed with PBS, labeled with 1 μM CFSE in PBS, co-cultured with BMDCs in 96-well U-bottom plates for 2 days, harvested, and washed with PBS. Proliferation was then evaluated via flow cytometry. OVA_257-264_-specific CD8^+^ T cells were co-cultured with BMDCs for 1 day, and cytokine secretion and intracellular cytokine concentrations were determined by ELISA and flow cytometry.

### Cytokine assay

Culture supernatants were analyzed by enzyme-linked immunosorbent assay (ELISA). The levels of various cytokines secreted by BMDCs, allogeneic CD4^+^ T cells, OVA-specific Th1 cells, and CD8^+^ T cells were measured by ELISA.

### Intracellular staining

CD8^+^ T cells were stained with biotin-anti-CD8 in the presence of anti-FcR (2.4G2), fixed with 4 % paraformaldehyde in PBS, permeabilized with 0.1 % saponin, and stained with FITC-anti-IFN-γ and PE-anti-CD8. CD8^+^ T cells were then gated and analyzed using a FACScanto II (BD Biosciences).

### Statistical analysis

The differences between groups are presented as the mean ± S.D. of three replicate experiments. The significance of differences was determined using the Student′s t-test. Probability values of < 0.05 were considered significant (P values are indicated as follows: ^*^ < 0.05, ^**^ < 0.01, ^***^ < 0.001, or ^+^ < 0.05, ^++^ < 0.01, ^+++^ < 0.001).

## Results

### HemoHIM is not cytotoxic to BMDCs

**Fig. 1:**
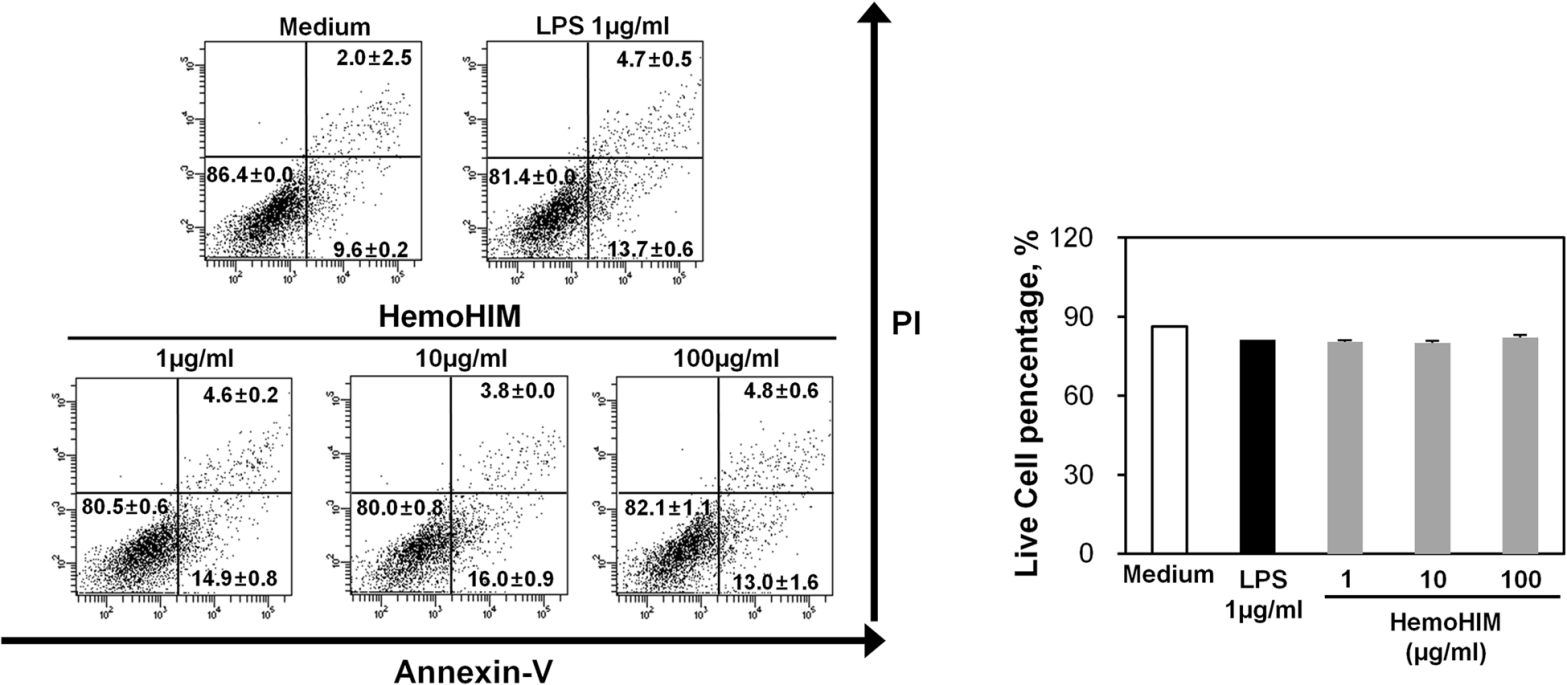
HemoHIM was not cytotoxic to BMDCs. BMDCs were treated with the indicated concentrations (1 μg/ml, 10 μg/ml and 100 μg/ml) of HemoHIM for 24 h, stained with Annexin V-FITC and PI, and analyzed by flow cytometry. The results are representative of three experiments

We investigated whether HemoHIM at different concentrations induces apoptosis in BMDCs. As shown in Fig. 1, HemoHIM did not induce BMDC apoptosis at any of the examined concentrations.

### HemoHIM induces the maturation of bone marrow-derived DCs

**Fig. 2:**
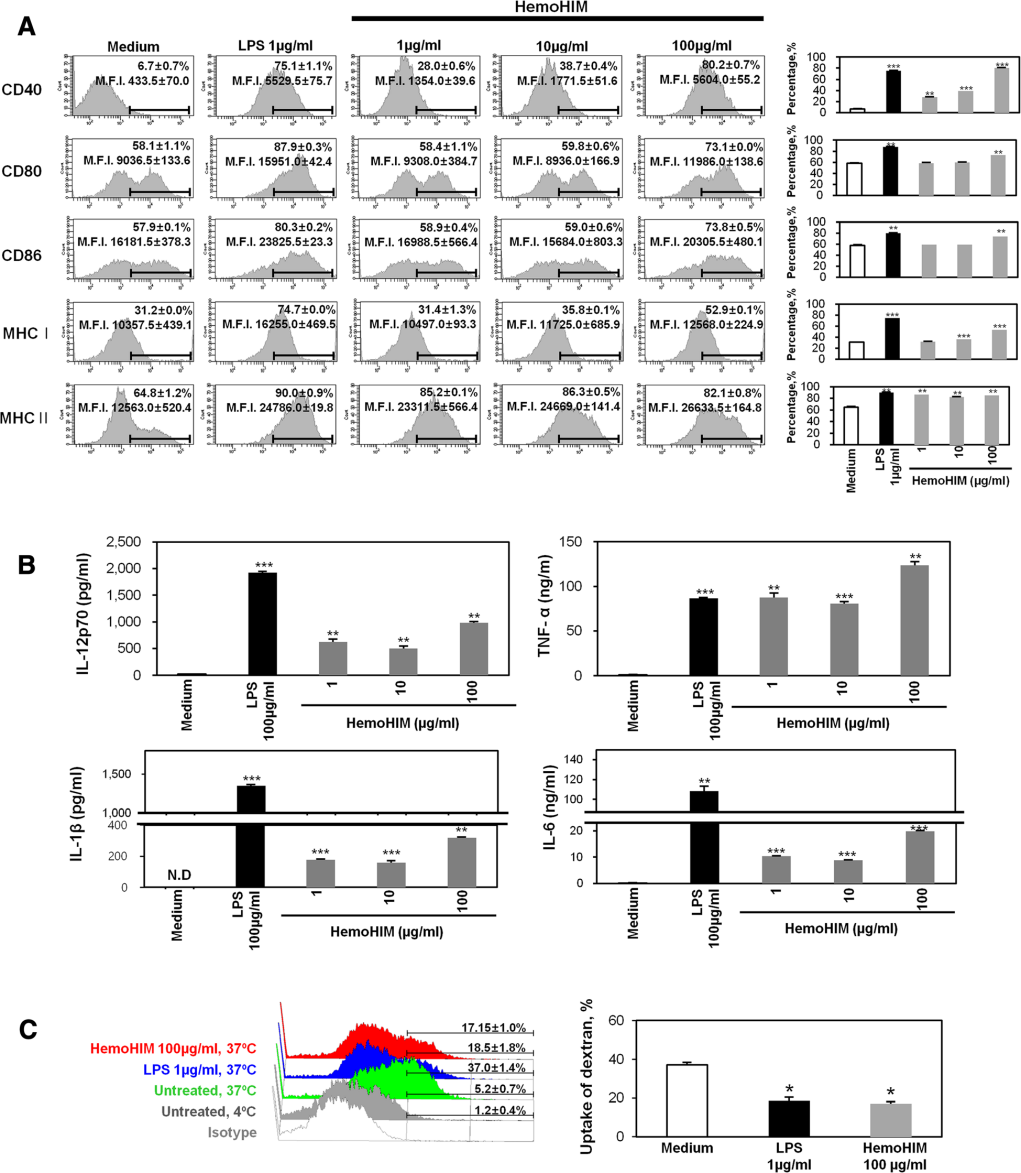
HemoHIM induced the phenotypic maturation of BMDCs. **a** BMDCs were treated with the indicated concentrations of LPS or HemoHIM for 24 h. Flow cytometry was used to assess the expressions of co-stimulatory molecules by CD11c^+^-gated BMDCs. The mean fluorescence intensities (M.F.I) and the percentages of positive cells are shown in each panel. **b** IL-1β, IL-6, IL-12p70, and TNF-α levels in HemoHIM-treated BMDCs were analyzed by ELISA. The results are representative of three experiments. ^*^, *p* < 0.05, ^**^, *p* < 0.01, and ^***^, *p* < 0.001 vs. untreated BMDCs. **c** Antigen uptake activities at 37 °C were assessed based on dextran-FITC uptake using flow cytometry. The percentages of dextran-FITC^+^ CD11c^+^ cells are indicated. The results are representative of three experiments. ^*^, *p* < 0.05, ^**^, *p* < 0.01, and ^***^, *p* < 0.001 vs. untreated BMDCs. Not detection (N.D) showed less than 10 pg/ml of cytokine secretion in this experiment

Flow cytometry was used to assess the expression of BMDC maturation markers, including CD40, CD80, CD86, and MHC I and II. In this experiment, BMDCs treated with LPS were used as a positive control. As shown in Fig. 2a, untreated BMDCs expressed CD40 (6.7 %), CD80 (58.1 %), CD86 (57.9 %), MHC I (31.2 %), and MHC II (64.8 %), where the listed percentages are indicate the percentage of cells expressing these markers. Compared to untreated BMDCs, BMDCs treated with LPS expressed higher levels of surface maturation markers: CD40 (75.1 %), CD80 (87.9 %), CD86 (80.3 %), MHC I (74.7 %), and MHC II (90.0 %). In addition, BMDCs treated with HemoHIM at concentrations of 1 μg/ml, 10 μg/ml and 100 μg/ml exhibited increased expression of surface markers. In particular, BMDCs treated with HemoHIM (100 μg/ml) expressed significantly higher levels of co-stimulatory molecules: CD40 (80.2 %), CD80 (73.1 %), CD86 (73.8 %), MHC I (52.9 %), and MHC II (85.2 %). These surface markers of BMDC maturation activate T cell responses via cell-to-cell interactions. However, this phenomenon requires interactions between surface markers and cytokines [1–10]. During maturation, BMDCs expressed higher levels of various co-stimulatory molecules and cytokines [1–10]. Cytokines, such as, IL-1β, IL-6, IL-12p70, and TNF-α, are important markers of BMDC maturation [1–10]. In particular, IL-12p70 is an important marker of DC maturation and stimulates Th1 cells [1–10]. In this experiment, BMDCs treated with LPS were used as a positive control. The secretion of IL-12p70 by BMDCs treated with a high concentration of HemoHIM (100 μg/ml) was higher than that of untreated BMDCs (Fig. 2b). The secretion of IL-1β, IL-6, and TNF-α by BMDCs exhibited similar patterns. Immature DCs have high antigen-uptake abilities, but after maturation, the cells lose this ability and mature into APCs [1–10]. Therefore, we examined whether mature DCs exhibited reduced antigen-uptake activity. BMDCs cultured in the presence or absence of HemoHIM or LPS were incubated with FITC-dextran for 24 h, and FITC-dextran-positive cells were then analyzed by flow cytometry. We found that the antigen uptake activity of HemoHIM-treated BMDCs was significantly lower than that of untreated BMDCs [(Fig. 2c) untreated: 30.3 % ±0.6, HemoHIM: 17.4 % ±1.4)]. Parallel experiments were performed at 4 °C to examine the non-specific uptake of FITC-dextran by BMDCs. At 4 °C, FITC-dextran was internalized by fewer than 5 % of DCs. Furthermore, HemoHIM-treated mature DCs exhibited a lower antigen uptake capacity for FITC-dextran than immature DCs, indicating that HemoHIM was more functionally active in mature DCs. These finding suggest that HemoHIM enhances the phenotypic and functional maturation of BMDCs.

### HemoHIM-treated bone marrow-derived DCs enhance allogeneic CD4^+^ T cell proliferation

**Fig. 3:**
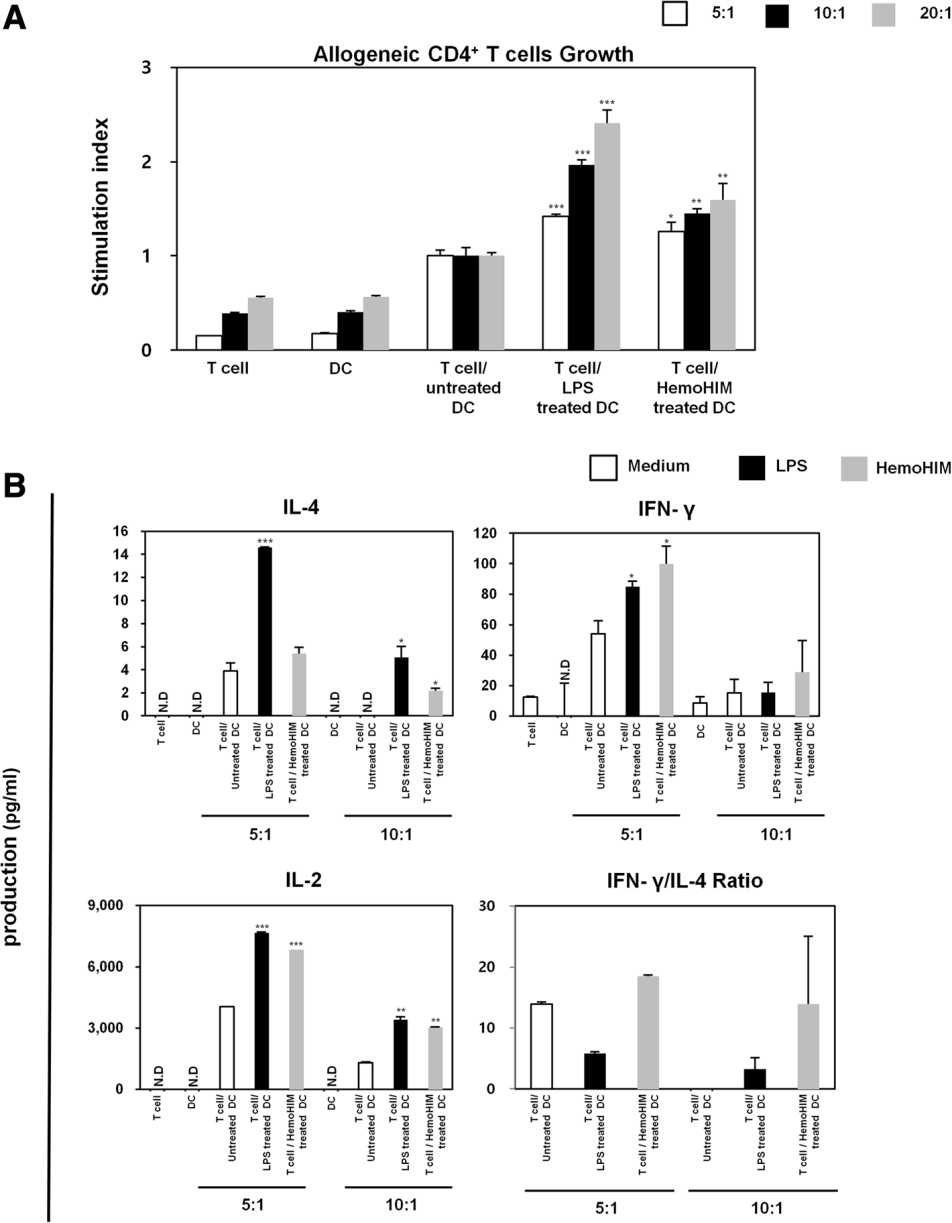
HemoHIM-treated BMDCs induced the activations of allogeneic CD4^+^ T cells derived from BALB/c mice. **a** CD4^+^ T cells were co-cultured for 48 h with C57BL/6-derived BMDCs that were treated with LPS (1 μg/ml) or HemoHIM (100 μg/ml). The proliferation of allogeneic CD4^+^ T cells was assessed using a Bromo-kit. **b** Culture supernatants were harvested after 48 h, and cytokine levels were measured by ELISA. The results are representative of three experiments. ^*^, *p* < 0.05, ^**^, *p* < 0.01, and ^***^, *p* < 0.001 vs. CD4^+^ T cells co-cultured with untreated BMDCs. Not detection (N.D) showed less than 10 pg/ml of cytokine secretion in this experiment

To examine the ability of HemoHIM-treated BMDCs to increase CD4^+^ and CD8^+^ T cell responses, we performed an allogeneic MLR assay using BALB/c CD4^+^ T cells. CD4^+^ T cells were co-cultured with untreated BMDCs, LPS-treated BMDCs, or HemoHIM-treated BMDCs. After co-culture for 2 days, CD4^+^ T cells co-cultured with HemoHIM-treated BMDCs were found to induce more CD4^+^ T cell proliferation than CD4^+^ T cells co-cultured with untreated BMDCs (Fig. 3a). To determine whether HemoHIM-treated BMDCs modulate cytokine secretion during allogeneic T cell responses, we measured IL-2, IFN-γ, and IL-4 levels in supernatants after co-culture for 2 days. HemoHIM-treated BMDCs co-cultured with CD4^+^ T cells secreted higher levels of IFN-γ, IL-4, and IL-2 than untreated BMDCs (Fig. 3b). In addition, the proliferation and cytokine secretion of CD4^+^ T cells increased in accordance with the T cell to DC ratio, and IFN-γ production increased more than IL-4 production (Fig. 3b). These results suggest that the activation and maturation of BMDCs by HemoHIM activates T cells and enhances Th1 responses.

### HemoHIM-treated bone marrow-derived DCs enhance antigen-specific CD4^+^ Th1 cell activation

**Fig. 4:**
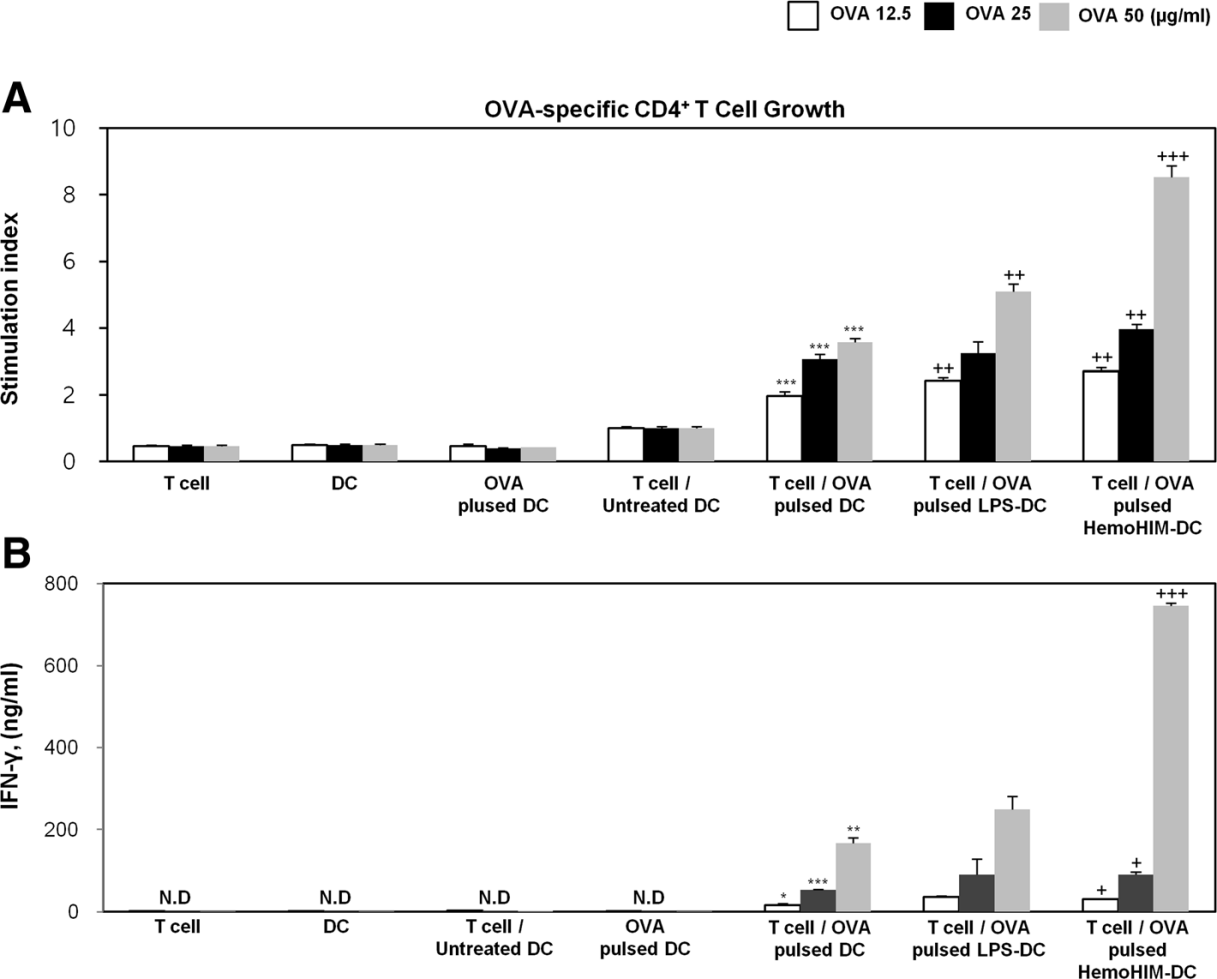
HemoHIM-treated BMDCs induced the activation of antigen-specific Th1 cells. **a** OVA-specific Th1 cells were co-cultured for 48 h with C57BL/6-derived BMDCs that were treated with LPS (1 μg/ml) or HemoHIM (100 μg/ml). The proliferation of OVA-specific Th1 cells was then assessed using a Bromo-kit. **b** Culture supernatants were harvested after 24 h, and cytokine levels were measured by ELISA. The results are representative of three experiments. ^*^, *p* < 0.05, ^**^, *p* < 0.01, and ^***^, *p* < 0.001 vs. OVA-specific T cells co-cultured with untreated BMDCs. ^+^, *p* < 0.05, ^++^, *p* < 0.01, and ^+++^, *p* < 0.001 vs. OVA-specific T cells co-cultured with OVA-pulsed BMDCs. Not detection (N.D) showed less than 10 pg/ml of cytokine secretion in this experiment

Next, we investigated whether HemoHIM treated BMDCs enhance antigen-specific CD4^+^ Th1 cell responses using OVA-specific Th1 cells. To measure the proliferation and cytokine secretion of OVA-specific Th1 cells, we co-cultured OVA-specific Th1 cells with or without treated BMDCs (LPS or HemoHIM). OVA-specific Th1 cells co-cultured with HemoHIM-treated BMDCs proliferated more than cells co-cultured with untreated BMDCs (Fig. 4a). Furthermore, OVA-specific Th1 cells co-cultured with HemoHIM-treated BMDCs secreted significantly more IFN-γ than cells co-cultured with untreated BMDCs (Fig. 4b), indicating that HemoHIM enhances antigen-specific Th1 cell responses induced by activated DCs.

### HemoHIM-treated bone marrow-derived DCs enhance OT-1 CD8^+^ T cell activation

**Fig. 5:**
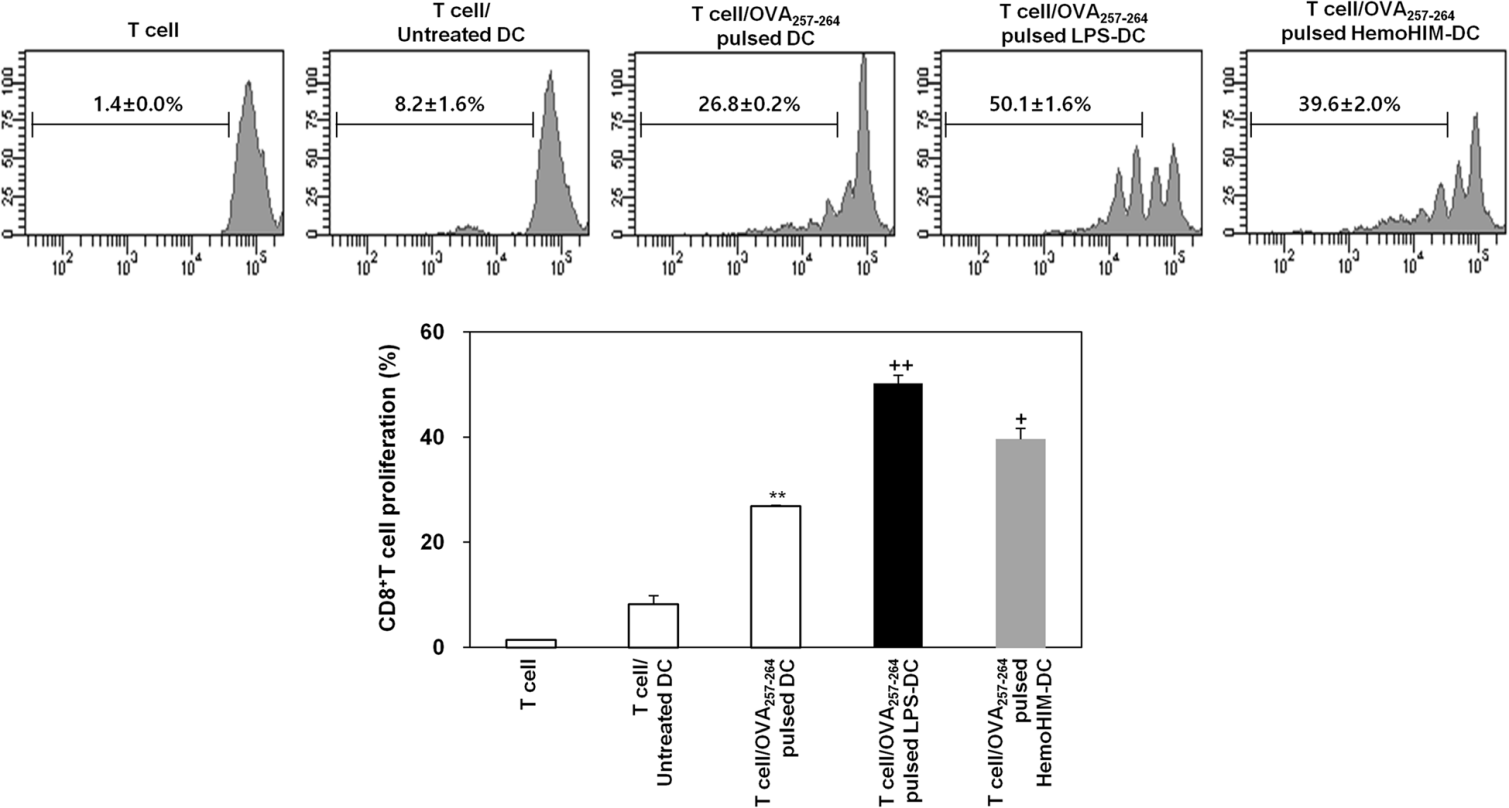
HemoHIM-treated BMDCs induced the activation of CD8^+^ T cells derived from C57BL/6 OT-1 mice. CD8^+^ T cells were stained with CFSE and co-cultured for 48 h with C57BL/6-derived BMDCs that were treated with LPS (1 μg/ml) or HemoHIM (100 μg/ml). The proliferation of CD8^+^ T cells was assessed by flow cytometry. The results are representative of three experiments. ^*^, *p* < 0.05, ^**^, *p* < 0.01, and ^***^, *p* < 0.001 vs. OVA_257-264_-specific T cells co-cultured with untreated BMDCs. ^+^, *p* < 0.05, ^++^, *p* < 0.01, and ^+++^, *p* < 0.001 vs. OVA_257-264_-specific T cells co-cultured with OVA_257-264_-pulsed BMDCs

**Fig. 6:**
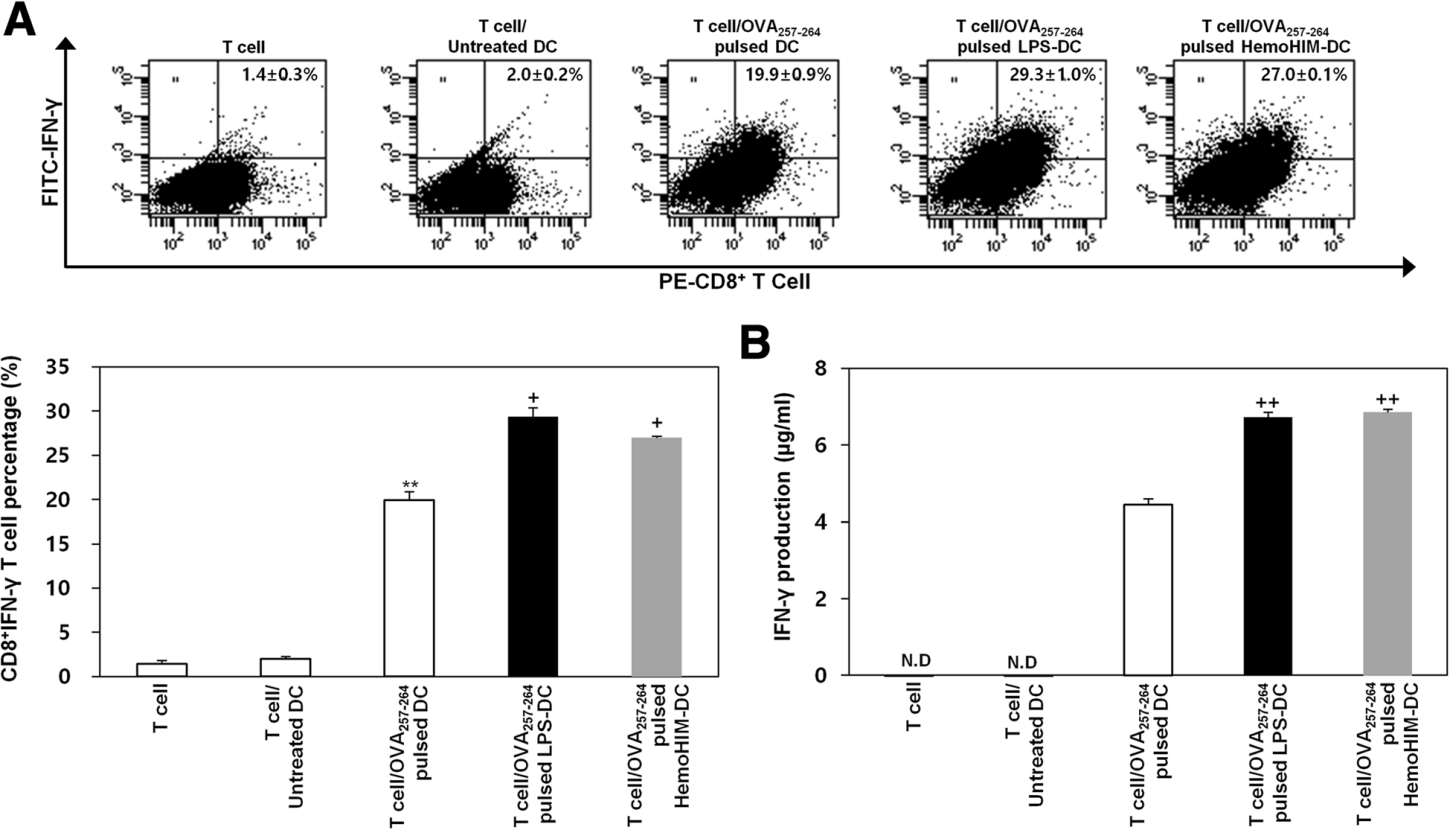
HemoHIM-treated BMDCs induced the activation of allogeneic CD8^+^ T cells derived from C57BL/6 OT-1 mice. CD8^+^ T cells were co-cultured for 24 h with C57BL/6-derived BMDCs that were treated with LPS (1 μg/ml) or HemoHIM (100 μg/ml). **a** Flow cytometry of intracellular IFN-γ staining in CD8^+^ T cells. **b** Culture supernatants were harvested after 24 h, and cytokine levels were measured by ELISA. The results are representative of three experiments. ^*^, *p* < 0.05, ^**^, *p* < 0.01, and ^***^, *p* < 0.001 vs. OVA_257-264_-specific T cells co-cultured with untreated BMDCs. ^+^, *p* < 0.05, ^++^, *p* < 0.01, and ^+++^, *p* < 0.001 vs. OVA_257-264_-specific T cells co-cultured with OVA_257-264_-pulsed BMDCs. Not detection (N.D) showed less than 10 pg/ml of cytokine secretion in this experiment

The presentation of captured antigens to cytotoxic CD8^+^ T cells is important for the induction of anti-tumor immunity. To examine the ability of HemoHIM-treated BMDCs to increase antigen-presentation to CD8^+^ T cells, we used OT-1 mice. CFSE-labeled CD8^+^ T cells were co-cultured with untreated BMDCs pulsed with OVA_257-264_ or treated BMDCs (LPS or HemoHIM) pulsed with OVA_257-264_. CD8^+^ T cells co-cultured with HemoHIM-treated BMDCs proliferated more than CD8^+^ T cells co-cultured with untreated BMDCs (Fig. 5). Furthermore, CD8^+^ T cells co-cultured with HemoHIM-treated BMDCs produced significantly more IFN-γ than those co-cultured with untreated BMDCs, presumably due to the secretion of intracellular cytokines (Fig. 6a) and the production (Fig. 6b) of cytokines. These results supported the notion that HemoHIM enhances CD8^+^ T cell responses by activating DCs.

### HemoHIM induces TLR4-mediated BMDC maturation

**Fig. 7:**
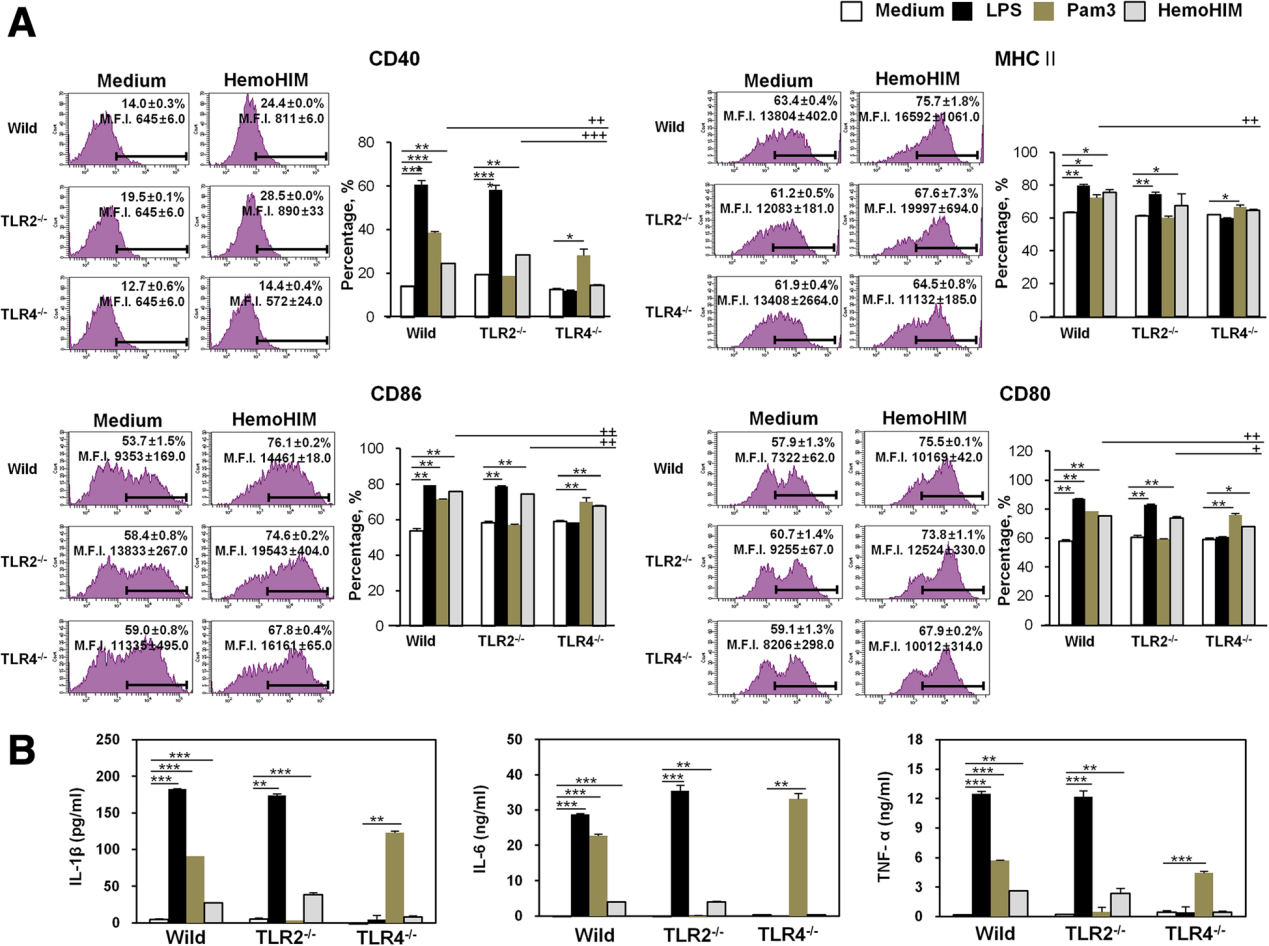
HemoHIM bound TLR4 but not TLR2 and induced BMDC activation. **a** Histograms of CD40, CD80, CD86, and MHC II expression by HemoHIM treated (100 μg/ml) CD11c-gated DCs derived from wild-type, TLR2-/-, or TLR4-/- mice. DCs derived from wild-type, TLR2-/- and TLR4-/- mice were treated with HemoHIM for 24 h. The percentages of positive cells are shown in each panel. **b** IL-1β, IL-6, and TNF-α production by HemoHIM-, LPS-, or Pam3-treated DCs derived from wild-type, TLR2-/-, or TLR4-/- mice were determined by ELISA. The results are representative of three experiments. ^*^, *p* < 0.05, ^**^, *p* < 0.01, and ^***^, *p* < 0.001 vs. untreated BMDCs. ^+^, *p* < 0.05, ^++^, *p* < 0.01, and ^+++^, *p* < 0.001 vs. HemoHIM-treated BMDCs from wild-type and TLR2^-/-^ mice

Associations between activated DCs and TLRs are critical for the tailoring of immune responses. We investigated whether TLRs on BMDCs recognized HemoHIM using TLR2^-/-^ and TLR4^-/-^ mice. To confirm that TLRs on BMDCs interact with HemoHIM, wild-type, TLR2^-/-^, or TLR4^-/-^ BMDCs were stimulated with HemoHIM for 24 h. Pam3 (a TLR2 agonist) and LPS (a TLR4 agonist) were used as positive controls. After stimulation for 24 h, we measured the expressions of the BMDCs maturation markers CD40, CD80, CD86, and MHC II and of various cytokines (i.e., IL-1β, IL-6, and TNF-α). As shown in Fig. 7a, untreated BMDCs from wild-type, TLR2^-/-^, and TLR4^-/-^ mice expressed similar levels of these markers and cytokines. However, HemoHIM-treated BMDCs from wild-type and TLR2^-/-^ mice exhibited higher expression of the investigated surface maturation markers than untreated wild-type or TLR2^-/-^ BMDCs [wild-type: CD40 (24.4 %), CD80 (75.5 %), CD86 (76.1 %), and MHC II (75.7 %); and TLR2^-/-^ : CD40 (28.5 %), CD80 (78.3 %), CD86 (74.6 %), and MHC II (67.6 %)]. In contrast, these effects were diminished in BMDCs from TLR4^-/-^mice, indicating that HemoHIM is an agonist of TLR4 in BMDCs [TLR4^-/-^ : CD40 (14.4 %) and CD80 (67.9 %)]. In contrast, the expression levels of CD80 and CD86 were significantly more reduced by HemoHIM in BMDCs from TLR4^-/-^ mice than in BMDCs from wild-type or TLR2^-/-^ mice [wild-type: CD80 (75.5 %) and CD86 (76.1 %); TLR2^-/-^ : CD80 (78.3 %) and CD86 (74.6 %); and TLR4^-/-^ : CD80 (67.9 %) and CD86 (67.8 %)]. In addition, cytokine production exhibited a pattern similar to that observed for surface molecules (Fig. 7b). These results demonstrate that HemoHIM induces TLR4-mediated BMDC maturation.

### HemoHIM enhances DCs maturation in vivo

**Fig. 8:**
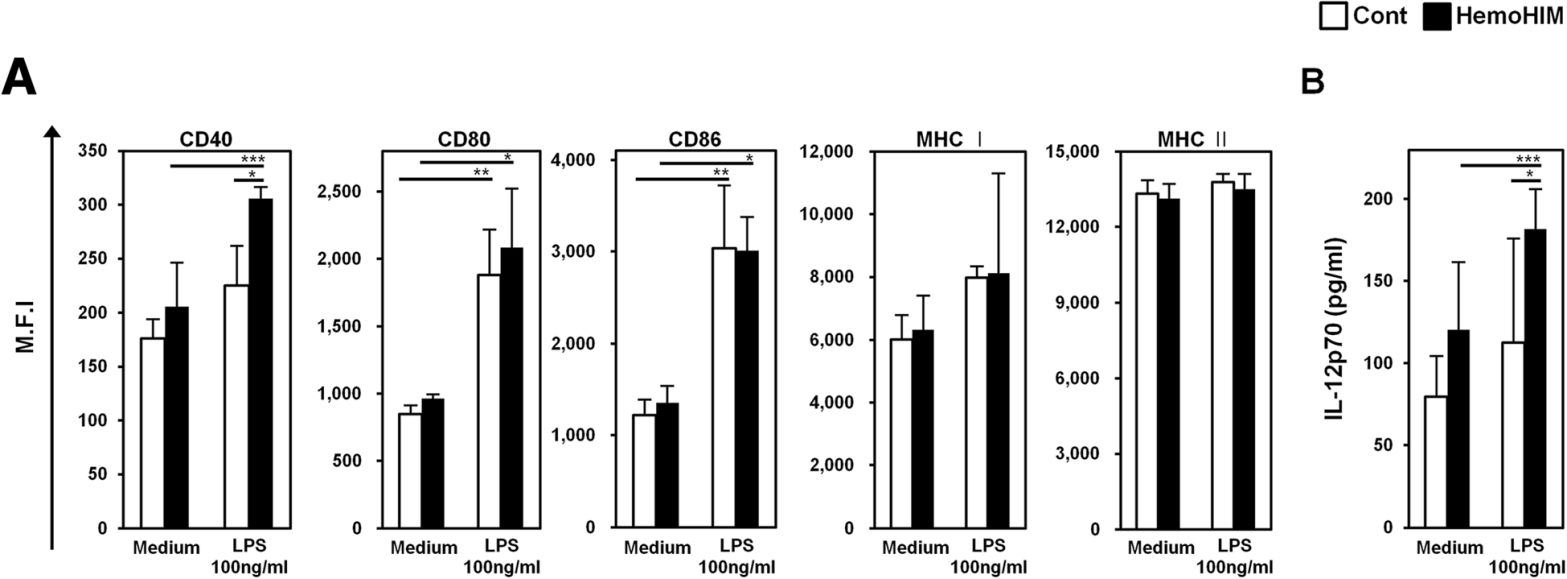
HemoHIM induced the phenotypic maturation and cytokine secretion of DCs in vivo. S-DCs was treated with the indicated concentrations of LPS for 24 h. **a** Flow cytometry was used to analyze the expressions of co-stimulatory molecules on CD11c^+^-gated s-DCs. Mean fluorescence intensities (M.F.I) of positive cells are shown for each panel. **b** IL-12p70 levels in HemoHIM-oral administration mice s-DCs were analyzed by ELISA. Results are representative of three experiments. ^*^, *p* < 0.05, ^**^, *p* < 0.01, and ^***^, *p* < 0.001 vs. untreated BMDCs. ^+^, *p* < 0.05, ^++^, *p* < 0.01, and ^+++^, *p* < 0.001 vs. LPS treated DCs of control group

**Fig. 9:**
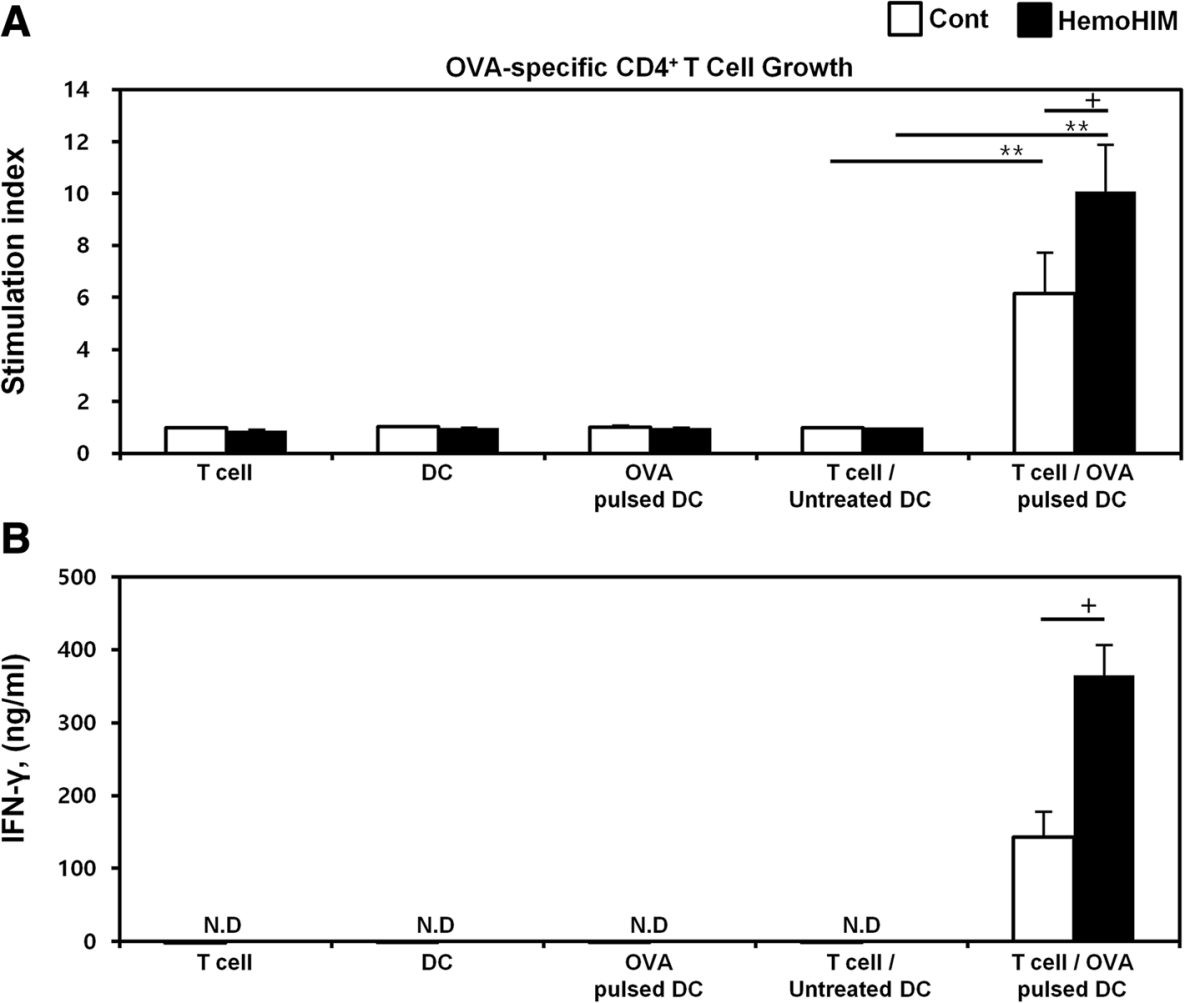
S-DCs of HemoHIM-oral administration mice induced the activation of antigen-specific T cells. OVA-specific T cells co-cultured for 48 h with s-DCs of each group pulsed with OVA (100 μg/ml). **a** The proliferation of OVA-specific T cells was assessed by flow cytometry. **b** Culture supernatants were harvested after 24 h, and IFN-γ levels were measured by ELISA. Results are representative of three experiments. ^+^, *p* < 0.05, ^++^, *p* < 0.01, and ^+++^, *p* < 0.001 vs. OVA-specific T cells co-cultured with untreated s-DC. ^*^, *p* < 0.05, ^**^, *p* < 0.01, and ^***^, *p* < 0.001 vs. OVA-specific T cells co-cultured with OVA-pulsed DCs of control group. Not detection (N.D) showed less than 10 pg/ml of cytokine secretion in this experiment

Through the results of the previous, we demonstrated that HemoHIM enhances the phenotypic and functional maturation of BMDCs and matured BMDCs by HemoHIM enhances antigen-specific T cell responses through in vitro. Likewise, we investigated whether HemoHIM enhanced function of s-DCs in vivo by oral administration HemoHIM. Mice were orally administered for 4 weeks. After 4 weeks, we isolated s-DCs in the spleen of mice each groups. After isolation, s-DCs were stimulated with LPS (100 ng/ml) for 24 h in order to maturation degree of s-DCs by LPS. After 24 h, we measured the expression of DC maturation markers such as CD40, CD80, CD86, MHC I, and MHC II and secretion cytokine IL-12p70. In the two groups, the results that s-DCs in the group with oral administration HemoHIM higher increased degree of maturation by LPS than s-DCs in the group with oral administration D.W through CD40 and CD80 among maturation markers. Also IL-12p70 secretion by s-DCs treated LPS was increased by administration HemoHIM group than by administration D.W group (Fig. 8a, b). Next we experimented that matured s-DCs of group with oral administered HemoHIM enhance antigen-specific T cell response with OVA-specific T cells than group with oral administered D.W. Proliferation and cytokine secretion of OVA specific T cell co-cultured OVA-pulsed s-DCs significantly increase by administration HemoHIM group than by administration D.W group. This result (Fig. 9) that HemoHIM enhanced functional maturation of s-DCs by in vivo.

## Discussion

Diseases are caused by the failure to induce appropriate immune responses to invading pathogens or tumor cells. Tumors are well-known sources of biological substances and release immunosuppressive factors to evade the host immune surveillance system [2, 46–48]. During neoplastic disease development, the body’s natural defenses are typically attenuated [49–51]. In particular, DCs that have infiltrated tumor tissues are known to exhibit reduced expressions of surface molecules and to produce fewer cytokines; this finding implies that tumor-derived factors impede DC maturation [49–51]. Furthermore, these effects appear to be maturation-dependent and to act on DC precursors rather than on mature DCs [2]. Recent studies indicate that DCs are the most potent immune cell vaccine candidate and that mature DCs are likely the best cell type for clinical use [2]. Immature DCs capture antigens generated by pathogens and tumors and then enter a maturation cascade. The resulting mature DCs exhibit elevated expression of co-stimulatory molecules and secrete more cytokines to promote immune synapse formation, antigen processing, antigen presentation to T cells, and T cell activation [10]. Therefore, DCs could potentially be used in vaccines for tumor-immunotherapy and to treat other diseases [52, 53]. In this study, we focused on the enhancement of DC activity. As mentioned above, HemoHIM has several biological activities and is non-cytotoxic, but it remained unknown whether HemoHIM modulates T cell responses in a DC-mediated manner. The present study demonstrates that HemoHIM interacts with BMDCs and induces DC maturation and that these events result in up-regulated expression of co-stimulatory molecules and the loss of the ability to take up antigens. Furthermore, HemoHIM-treated BMDCs were found to secrete considerably more cytokines (i.e., IL-1β, IL-6, IL-12p70, and TNF-α) than untreated cells. Of the cytokines secreted by DCs, IL-12p70 is most important in the context of DC maturation because this ctokine induces the differentiation of naïve CD4^+^ T cells into Th1 cells [9, 10]. In the present study, HemoHIM was found to induce the phenotypic and functional maturation of DCs, indicating that this treatment has immunoadjuvant potential, as suggested previously [1–10]. After maturation, DCs interact with T cells using co-stimulatory molecules and cytokines. The present study demonstrates that DCs that mature in response to HemoHIM induce greater proliferation and cytokine secretion by allogeneic CD4^+^ T cells and OVA-specific Th1 cells than their untreated counterparts. In particular, IFN-γ secretion was more enhanced than IL-4 secretion. It is also possible HemoHIM induces Th1 response because IL-12p70 is associated with Th1 activation. In addition, in the present study, HemoHIM-treated BMDCs enhanced CD8^+^ T cell responses in OT-1 mice. These results demonstrated that HemoHIM-treated BMDCs induce T cell activation and suggest that HemoHIM-treated BMDCs may more effectively induce T cell responses against antigens.

TLR ligands are promising candidate immune stimulatory adjuvants for tumor-therapy [12] and have been reported to be expressed on APCs, including immune cells, and to be required for immune responses [54–56]. Moreover, TLR4 has been reported to induce Th1 responses in studies of immune adjuvants for tumor-therapy [54–56]. In the present study, co-stimulatory molecule expression and the amount of cytokines secreted by HemoHIM-treated BMDCs from TLR4^-/-^ mice were decreased, while cytokine secretion by BMDCs from wild-type and HemoHIM-treated TLR2^-/-^ mice were increased. Previous studies of a variety of immunoadjuvant plant extracts that function via DC-mediated immune responses have focused on TLR4 agonists [10, 57, 58]. However, HemoHIM is an herbal mixture, and perhaps only one of its components is recognized by TLR4.

The present study demonstrates that HemoHIM induces the phenotypic and functional maturation of DCs, which initiate T cell responses, including specific-CD8^+^ T cell responses and specific-CD4 Th1 responses, in a TLR-4 mediated manner. Also, we proved that HemoHIM enhances functional maturation of s-DCs by in vivo. HemoHIM in combination with cisplatin was previously reported to have anti-cancer effects in vitro and in vivo [31], and the present study suggests that these effects are due to the enhancement of T cell responses when DCs are treated with HemoHIM. Accordingly, our findings indicate that HemoHIM is a potential tumor-therapy that enhances DC-based T cell responses.

## Conclusions

In summary, our study demonstrated that HemoHIM induces TLR4-mediated BMDCs maturation. Furthermore, we showed the antigen-presenting ability that HemoHIM-treated mature BMDCs increase CD4^+^ and CD8^+^ T cell responses by in vitro. Therefore, HemoHIM has the potential dendritic cell-based immunoadjuvant.

## Abbreviations

Abs: antibodies
APC: antigen presenting cell
BMDC: bone marrow-derived dendritic cell
BrdU: 5-Bromo-2′-Deoxy-Uridine
CCR: chemokine receptor 7
CD: cluster of differentiation
CFA: complete Freund’s adjuvant
CFSE: carboxyfluorescein succinimidyl ester
DC: dendritic cell
DW: distilled water
ELISA: enzyme-linked immunosorbent assay
FACS: fluorescence-activated cell sorting
FBS: fetal bovine serum
FITC: fluorescein isothiocyanate
GM-CSF: granulocyte-macrophage colony-stimulating facto
IFA: incomplete Freund’s adjuvant
IFN: interferon
IL: interleukin
ip: intraperitoneal injection
LPS: lipopolysaccharide
MHC: major histocompatibility complex
MLR: mixed-lymphocyte reaction
MMC: mitomycin C
OVA: ovalbumin
Pam3: pam3csk4
PBS: phosphate buffered saline
PE: phycoerythrin
PI: propidium iodide
S-DC: spleen-dendritic cell
TCR: T-cell receptor
Th: helper T cell
TLR: toll-like receptor
TNF: tumor necrosis factor
